# Supplementary material for: Single-cell transcriptomics reveals a new dynamical function of transcription factors during embryonic hematopoiesis
Source: eLife. 2018 Mar 20;7:e29312. doi: 10.7554/eLife.29312 (PMC5860872; doi:10.7554/eLife.29312)
Supplement: Supplementary file 1. — The genes are classified by categories. A gene can belong to several categories. [file elife-29312-supp1.docx]

| Gene classification | Gene names |
| --- | --- |
| Endothelial genes | *Cdh5, Cldn5, Eng, Erg, Esam, Fbn1, Gpr126, Kdr, Meis2, Notch1, Npr1, Pcdh12, Ptprb, Ptprm, Ramp2, Sox7, Tek* |
| Hematopoietic genes | *Epo, Epor, Gata1, Gata2, Gfi1, Gfi1b, Hbb-bh1, Itga2b, Itgam, Itgb3, Kit, Lmo2, Lyl1, Mpo, Myb, Ptprc, Runx1, Sfpi1, Sla, Tal1, Thpo* |
| Gene Expression Regulators | *Erg, Fli1, Gata1, Gata2, Gfi1, Gfi1b, Lmo2, Lyl1, Meis2, Myb, Runx1, Samd4, Sfpi1, Snai1, Snai2, Sox7, Tal1* |
| TGFbeta-BMP pathway related genes | *Acvr1, Acvr1b, Acvr2a, Acvr2b, Acvrl1, Bmp4, Bmpr1a, Bmpr2, Eng, Smad1, Smad2, Smad3, Smad4, Smad5, Smad6, Smad7, Smad9, Tgfb1, Tgfb2, Tgfb3, Tgfbr1, Tgfbr2* |
| Epithelial Mesenchymal Transition related gene | *Acta2, Cdh2, Serpine1, Snai1, Snai2, Tgfb1, Tgfb2, Tgfb3, Tgfbr1, Tgfbr2, Ctnnb1* |
| Cell surface markers | *Acvr1, Acvr1b, Acvr2a, Acvr2b, Acvrl1, Bmpr1a, Bmpr2, Cdh2, Cdh5, Cldn5, Eng, Enpp1, Epor, Esam, Flrt2, Gdpd5, Gpr126, Gria4, Lgr5, Itga2b, Itgam, Itgb3, Kdr, Kit, Met, Notch1, Nrp1, Pcdh12, Pecam1, Ptprb, Ptprc, Ptprm, Ramp2, Tek, Tgfbr1, Tgfbr2* |
| Secreted molecules | *Bmp4, Col4a2, Col4a5, Epo, Pdzd2, Serpine 1, Tgfb1, Tgfb2, Tgfb3, Thpo* |
| Signaling | *Adcy4, Atp2a3, Cacna2d1, Ctnnb1, Dcaf12l1, Dpysl3, Lat, Ppp1r16b, Plcd1, Sash1, Sla, She, Smad1, Smad2, Smad3, Smad4, Smad5, Smad6, Smad7, Smad9* |
| Cytoskeleton | *Acta2, Eps8, Fbn1, Lad1, Myom1, Palld* |
| Metabolism | *Fmo1, Upp1* |
| Reference gene | *Ppia* |

**Supplementary file 1:**

**Description of the genes used for single cell quantitative RT-PCR.** The genes are classified by categories. A gene can belong to several categories.
