# Supplementary material for: Single-cell transcriptomics reveals a new dynamical function of transcription factors during embryonic hematopoiesis
Source: eLife. 2018 Mar 20;7:e29312. doi: 10.7554/eLife.29312 (PMC5860872; doi:10.7554/eLife.29312)
Supplement: Supplementary file 11. — For each figure panel, the type of experiments is described as well as the number of times they were carried out. [file elife-29312-supp11.docx]

| FIGURE | TYPE OF EXPERIMENTS | NUMBER OF EXPERIMENTS |
| --- | --- | --- |
| 1A | Isolation of mouse embryos, dissection of AGM and yolk sac, FACS sorting and Single-cell q-RT-PCR | One experiment was done per time point. For each experiment, AGM and Yolk Sac cells were always isolated from the same litter of mouse embryos.  At E9, AGM (62 cells) and Yolk Sac (89 cells) cells were isolated from one litter of seven mouse embryos (14 to 21 somite pairs) (**n=1**).  At E10.5, AGM (85 cells) and Yolk Sac (83 cells) cells were isolated from two litters of seven and six mouse embryos (33 to 37 somite pairs) (**n=1**).  At E11, AGM (78 cells) and Yolk Sac (92 cells) cells were isolated from one litter of nine mouse embryos (39 to 43 somite pairs) (**n=1**). |
| 1B | Same as in Figure 1A | All 59 Gfi1^-/-^ Gfi1b^-/-^ yolk sac cells are from one mouse embryo (**n=1**).  All other cells are the same as in Figure 1A. |
| 1D | Differentiation of ESCs (BL-CFC culture), FACS sorting and Single-cell q-RT-PCR | All 174 cells come from one BL-CFC culture (**n=1**). |
| 1- Figure Supplement 1A | Isolation of mouse embryos, dissection of AGM, Flow cytometry analysis | Same as in Figure 1A |
| 1- Figure Supplement 1B | Same as in Figure 1 - Figure Supplement 1A followed by Single-cell q-RT-PCR | Same as in Figure 1A |
| 1- Figure Supplement 2A | Isolation of mouse embryos, dissection of yolk sac, Flow cytometry analysis | Same as in Figure 1A |
| 1- Figure Supplement 2B | Same as in Figure 1 - Figure Supplement 2A followed by Single-cell q-RT-PCR | Same as in Figure 1A |
| 1- Figure Supplement 3 | Same as in Figure 1A | Same as in Figure 1A |
| 1- Figure Supplement 4 | Same as in Figure 1A | Same as in Figure 1A |
| 1- Figure Supplement 5A | Isolation of mouse embryos, dissection of yolk sac, Flow cytometry analysis | Same as in Figure 1B |
| 1- Figure Supplement 5B | Isolation of mouse embryos, dissection of yolk sac, FACS sorting and Single-cell q-RT-PCR | Same cells as in Figure 1B without the cells as shown in Figure 1A |
| 1- Figure Supplement 6A | Same as in Figure 1D | Same as in Figure 1D |
| 1- Figure Supplement 6B | Same as in Figure 1B | Same as in Figure 1B |
| 1- Figure Supplement 6C | Same as in Figure 1D | Same as in Figure 1D |
| 2 | Culture of ESCs with or without doxycycline, Protein extraction, Western Blot | The results are from one experiment (**n=1**). |
| 3A | Differentiation of ESCs (BL-CFC culture), Flow cytometry analysis | The two indicated cell lines have been differentiated independently three times and Flow cytometry analysis was performed for each differentiation. The plots show one representative experiment (**n=3**). |
| 3B | Differentiation of ESCs (BL-CFC culture), Time lapse microscopy | The graphs are derived from three sets of microscopic images of one representative well of BL-CFC culture with or without dox for each cell line (**n=1**). |
| 3C | Differentiation of ESCs (BL-CFC culture with or without dox), Apoptosis assay | The two indicated cell lines have been differentiated independently three times and the apoptosis assay was performed for each differentiation. The results are from three independent experiments (**n=3**). |
| 3D | Differentiation of ESCs (BL-CFC culture with or without dox), Proliferation assay | The two indicated cell lines have been differentiated independently three times and the proliferation assay was performed for each differentiation. The results are from three independent experiments (**n=3**). |
| 3E | Differentiation of ESCs (BL-CFC culture with or without dox), CFU assay | The indicated cell line has been differentiated independently four times and the CFU assay was performed for each differentiation. The results are from four independent experiments (**n=4**). |
| 3- Figure Supplement 1 | Differentiation of ESCs (BL-CFC culture with or without dox), Microscopy | Same as in Figure 3A. The images are derived from one representative experiment (**n=3**). |
| 3 - Figure Supplement 2A | Differentiation of ESCs (BL-CFC culture), Flow cytometry analysis | The i8TFs cell line has been differentiated independently three times and Flow cytometry analysis was performed for each differentiation. The plot represents one representative experiment (**n=3**). |
| 3 - Figure Supplement 2B | Differentiation of ESCs (BL-CFC culture), FACS sorting, Single-cell q-RT-PCR | All 93 cells are from one experiment (**n=1**). |
| 3 - Figure Supplement 2C | Differentiation of ESCs (BL-CFC culture), FACS sorting, Hemogenic Endothelium culture with or without dox, Flow cytometry analysis | The experiment has been performed three times independently. The plots show one representative experiment (**n=3**). |
| 3 - Figure Supplement 2D | Differentiation of ESCs (BL-CFC culture), FACS sorting, Hemogenic Endothelium culture with or without dox, CFU assay | The i8TF cell line has been differentiated independently two times and the CFU assay was performed for each differentiation. The results are from two independent experiments (**n=2**). |
| 3 - Figure Supplement 3A | Differentiation of ESCs (BL-CFC culture), FACS sorting, Hemogenic Endothelium culture with or without dox, Microarrays | The empty cell line has been differentiated independently three times and the microarrays analysis was performed for each differentiation. The results are from three independent experiments (**n=3**). |
| 3 - Figure Supplement 3B | Differentiation of ESCs (BL-CFC culture), FACS sorting, Hemogenic Endothelium culture with or without dox, Microarrays | The i8TF cell line has been differentiated independently three times and the microarrays analysis was performed for each differentiation. The results are from three independent experiments (**n=3**). |
| 3 - Figure Supplement 4 | Same as in Figure 3 - Figure Supplement 3B | Same as in Figure 3 - Figure Supplement 3B |
| 3 - Figure Supplement 5B | Culture of the eight ESC lines with or without doxycycline, Protein extraction, Western Blot | For each cell line, the results are from one experiment (**n=1**). |
| 3 - Figure Supplement 5C | Differentiation of the eight ESC lines (BL-CFC culture with or without dox), Flow cytometry analysis | The eight indicated cell lines have been differentiated independently three times and Flow cytometry analysis was performed for each differentiation. For each cell line, the plots show one representative experiment (**n=3**) |
| 3 - Figure Supplement 5D | Differentiation of the ten ESC lines (BL-CFC culture with or without dox), Flow cytometry analysis | The ten cell lines have been differentiated independently three times and the flow cytometry analysis was performed for each differentiation. The graphs summarise the results of three independent experiments (**n=3**). |
| 4 | Differentiation of the ten ESC lines (BL-CFC culture with or without dox), FACS sorting, Single-cell q-RT-PCR | All 854 cells come from one BL-CFC culture for each of the ten ESC lines. Each line had cells from “no dox” and “with dox” combined on one Fluidigm 96.96 dynamic array IFC.  **Empty** Cell line: 42 cells no dox and 45 cells with dox (**n=1**).  **i8TFs** line: 41 cells no dox and 45 cells with dox (**n=1**).  **iCbfb** Cell line: 46 cells no dox and 46 cells with dox (**n=1**).  **iErg** Cell line: 38 cells no dox and 36 cells with dox (**n=1**).  **iFli1** Cell line: 45 cells no dox and 45 cells with dox (**n=1**).  **iGata2** Cell line: 42 cells no dox and 46 cells with dox (**n=1**).  **iLmo2** Cell line: 41 cells no dox and 41 cells with dox (**n=1**).  **iLyl1** Cell line: 46 cells no dox and 43 cells with dox (**n=1**).  **iRunx1** Cell line: 41 cells no dox and 38 cells with dox (**n=1**).  **iTal1** Cell line: 42 cells no dox and 45 cells with dox (**n=1**). |
| 4 - Figure Supplement 1 | Same as in Figure 4 | Same as in Figure 4 |
| 4 - Figure Supplement 2 | Differentiation of ESCs (BL-CFC culture with or without dox), Single-cell RNA sequencing (Wafergen ICELL8 platform) | The two indicated cell lines have been differentiated once and single cells were isolated from one BL-CFC culture (**n=1**). |
| 5A | Differentiation of ESCs (BL-CFC culture), Flow cytometry analysis | The indicated cell line has been differentiated independently three times and Flow cytometry analysis was performed for each differentiation. The plots show one representative experiment (n=3). |
| 5B | Same as in Figure 5A | Same as in Figure 5A. The graph summarizes the results of the three independent experiments (**n=3**). |
| 5C | Isolation of mouse embryos, FACS sorting and Single-cell q-RT-PCR | Endo, Pre-HSPCs and HSPCs are the same as in Figure 1A.  i8TFs cells come from one BL-CFC culture (see Figure 4A).  The 43 Pro-HSCs and 41 Pre-HSCs type I cells are from two litters of seven and three mouse embryos (31 to 32 somite pairs) (**n=1**). |
| 5 - Figure Supplement 1A | Isolation of mouse embryos, dissection of AGM, Flow cytometry analysis | The FACS plots show AGM cells from two litters of seven and three mouse embryos (31 to 32 somite pairs) (**n=1**). |
| 5 - Figure Supplement 1B | Isolation of mouse embryos, dissection of AGM, FACS sorting, Single-cell q-RT-PCR | The 43 Pro-HSCs and 41 Pre-HSCs type I cells are isolated from two litters of seven and three mouse embryos (31 to 32 somite pairs) (**n=1**). |
| 5 - Figure Supplement 2 | Same as in Figures 1, 4 and 5 | Same as in Figures 1, 4 and 5 |
| 6A | Differentiation of ESCs (BL-CFC culture), FACS sorting, Hemogenic Endothelium culture with or without dox, Single-cell RNA sequencing (Fluigdim C1 platform) | The i8TFs cell line has been differentiated independently two times and Single-cell RNA sequencing analysis was performed for each differentiation. The results combined single cells from two independent experiments (**n=2**). |
| 6C | Culture of ESCs, Transfection, Replacement of medium with or without doxycycline, Luciferase Reporter Assays | The i8TFs ES cell line has been transfected and treated with doxycycline in three independent experiments. The graph summarizes the results of three independent experiments (**n=3**). |
| 6 - Figure Supplement 1 | Differentiation of ESCs (BL-CFC culture), FACS sorting, Hemogenic Endothelium culture with or without dox, Single-cell RNA sequencing (Fluigdim C1 platform) | Same samples as in Figure 6A |
| 8B | Differentiation of ESCs (BL-CFC culture), Flow cytometry analysis | The two indicated cell lines have been differentiated independently three times and Flow cytometry analysis was performed for each differentiation. The plots show one representative experiment (**n=3**). |
| 8C | Differentiation of ESCs (BL-CFC culture), Time lapse microscopy | The graphs are derived from three sets of microscopic images of one representative well of BL-CFC culture with or without dox for each cell line (**n=1**). |
| 8D | Differentiation of ESCs (BL-CFC culture), FACS sorting, Hemogenic Endothelium culture with or without dox, Microscopy | The experiment has been replicated once. The images are derived from one representative Hemogenic Endothelium culture with or without dox for each cell line (**n=2**). |
| 8E | Differentiation of the two ESC lines (BL-CFC culture with or without dox), FACS sorting, Single-cell q-RT-PCR | All 171 cells come from one BL-CFC culture for each of the two ESC lines. Each line had cells from “no dox” and “with dox” combined on one Fluidigm 96.96 dynamic array IFC.  **i5TFs** line: 41 cells no dox and 43 cells with dox (**n=1**).  **I6TFs** Cell line: 43 cells no dox and 44 cells with dox (**n=1**). |
| 8 - Figure Supplement 1A | Differentiation of ESCs (BL-CFC culture), FACS sorting, Hemogenic Endothelium culture with or without dox, Time lapse microscopy | The graphs are derived from three sets of microscopic images of one representative Hemogenic Endothelium culture with or without dox for each cell line (**n=1**). |
| 8 - Figure Supplement 1B | Differentiation of the two ESC lines (BL-CFC culture with or without dox), FACS sorting, Single-cell q-RT-PCR | Same as in Figure 8E |

**Supplementary file 11: Table summarizing the experiments done in the manuscript.** For each figure panel, the type of experiments is described as well as the number of times they were done.
