# Supplementary material for: Single-cell transcriptomics reveals a new dynamical function of transcription factors during embryonic hematopoiesis
Source: eLife. 2018 Mar 20;7:e29312. doi: 10.7554/eLife.29312 (PMC5860872; doi:10.7554/eLife.29312)
Supplement: Transparent reporting form [file elife-29312-transrepform.docx]

***eLife’s* transparent reporting form**

We encourage authors to provide detailed information *within their submission* to facilitate the interpretation and replication of experiments. Authors can upload supporting documentation to indicate the use of appropriate reporting guidelines for health-related research (see [EQUATOR Network](http://www.equator-network.org/%20)), life science research (see the [BioSharing Information Resource](https://biosharing.org/)), or the [ARRIVE guidelines](http://www.plosbiology.org/article/info:doi/10.1371/journal.pbio.1000412) for reporting work involving animal research. Where applicable, authors should refer to any relevant reporting standards documents in this form.

If you have any questions, please consult our Journal Policies and/or contact us: [editorial@elifesciences.org](mailto:editorial@elifesciences.org).

**Sample-size estimation**

- You should state whether an appropriate sample size was computed when the study was being designed
- You should state the statistical method of sample size computation and any required assumptions
- If no explicit power analysis was used, you should describe how you decided what sample (replicate) size (number) to use

Please outline where this information can be found within the submission (e.g., sections or figure legends), or explain why this information doesn’t apply to your submission:

Experiments have been designed according to pre-existing and published research papers in this field. No explicit power calculations have been performed.

For all primary research questions in this study the choice of the number of replicates was guided by comparable studies in which significant results were obtained (see Vargel et al. *Sci Rep* 2016 PMID: 26891705, Kanki et al. *NAR* 2017 PMID: 28334937 and Tsang et al. *Genome Biol* 2015 PMID: 26387834).

**Replicates**

- You should report how often each experiment was performed
- You should include a definition of biological versus technical replication
- The data obtained should be provided and sufficient information should be provided to indicate the number of independent biological and/or technical replicates
- If you encountered any outliers, you should describe how these were handled
- Criteria for exclusion/inclusion of data should be clearly stated
- High-throughput sequence data should be uploaded before submission, with a private link for reviewers provided (these are available from both GEO and ArrayExpress)

Please outline where this information can be found within the submission (e.g., sections or figure legends), or explain why this information doesn’t apply to your submission:

All information related to replicates are listed in the material and methods, subsection called Quantification and Statistical Analysis.

**Statistical reporting**

- Statistical analysis methods should be described and justified
- Raw data should be presented in figures whenever informative to do so (typically when N per group is less than 10)
- For each experiment, you should identify the statistical tests used, exact values of N, definitions of center, methods of multiple test correction, and dispersion and precision measures (e.g., mean, median, SD, SEM, confidence intervals; and, for the major substantive results, a measure of effect size (e.g., Pearson's r, Cohen's d)
- Report exact p-values wherever possible alongside the summary statistics and 95% confidence intervals. These should be reported for all key questions and not only when the p-value is less than 0.05.

Please outline where this information can be found within the submission (e.g., sections or figure legends), or explain why this information doesn’t apply to your submission:

All information related to statistical reporting are listed in the material and methods, subsection called Quantification and Statistical Analysis.

(For large datasets, or papers with a very large number of statistical tests, you may upload a single table file with tests, Ns, etc., with reference to sections in the manuscript.)

**Group allocation**

- Indicate how samples were allocated into experimental groups (in the case of clinical studies, please specify allocation to treatment method); if randomization was used, please also state if restricted randomization was applied
- Indicate if masking was used during group allocation, data collection and/or data analysis

Please outline where this information can be found within the submission (e.g., sections or figure legends), or explain why this information doesn’t apply to your submission:

Samples were allocated based on the treatment applied (- or + doxycycline). No masking was used during group allocation, data collection and/or data analysis.

**Additional data files (“source data”)**

- We encourage you to upload relevant additional data files, such as numerical data that are represented as a graph in a figure, or as a summary table
- Where provided, these should be in the most useful format, and they can be uploaded as “Source data” files linked to a main figure or table
- Include model definition files including the full list of parameters used
- Include code used for data analysis (e.g., R, MatLab)
- Avoid stating that data files are “available upon request”

Please indicate the figures or tables for which source data files have been provided:

All our single cell qPCR raw data have been added as supplementary files:

Supplementary file 2 (related to Figure 1)

Supplementary file 3 (related to Figure 1D)

Supplementary file 4 (related to Figure 3 - Figure Supplement 2B)

Supplementary file 6 (related to Figure 4)

Supplementary file 7 (related to Figure 5)

Supplementary file 8 (related to Figure 5 - Figure Supplement 2)

Supplementary file 10 (related to Figure 8 and Figure 8 - Figure Supplement 1)

The single cell RNA sequencing and microarray data used for the graphs in the Figure 4 - Figure Supplement 2, Figure 6, Figure 3 - Figure Supplement 3 & 4 have been deposited in GEO (accession number: GSE96986).
